# Co-Amorphous Solid Dispersion System for Improvement in Dissolution Profile of *N*-(((1*r*,4*r*)-4-((6-fluorobenzo[*d*]oxazol-2-yl)amino)cyclohexyl)methyl)-2-methylpropane-2-sulfonamide as a Neuropeptide Y5 Receptor Antagonist

**DOI:** 10.3390/pharmaceutics16101293

**Published:** 2024-10-02

**Authors:** Hironori Tanaka, Hiroshi Ueda

**Affiliations:** 1 Formulation R&D Laboratory, Shionogi & Co., Ltd., Amagasaki 660-0813, Hyogo, Japan; hironori.tanaka@shionogi.co.jp; 2 Analysis and Evaluation Laboratory, Shionogi & Co., Ltd., Toyonaka 561-0825, Osaka, Japan

**Keywords:** amorphous, co-amorphous, co-amorphous solid dispersion, solid dispersion

## Abstract

**Background/Objectives:** Brick dust molecules exhibit high melting points and ultralow solubility. Overcoming this solubility issue is challenging. Previously, we formulated a co-amorphous system for a neuropeptide Y5 receptor antagonist (NP) as a brick dust drug using sodium taurocholate (ST) to improve its dissolution profile. In this study, we have designed a ternary amorphous system involving polymer addition to further improve a co-amorphous system. **Methods:** The amorphous samples were prepared by the ball milling. The thermal and spectroscopic analyses were performed, and the isothermal crystallization and dissolution profiles were evaluated. **Results:** The ball milling of NPs, ST, and each of the three types of polymers successfully converted crystalline NPs to amorphous NPs. Thermal analysis confirmed the formation of a single amorphous phase. The infrared spectra revealed a specific interaction between an NP and ST in the co-amorphous system. Moreover, the intermolecular interactions of NP-ST were maintained in the ternary amorphous systems, suggesting the miscible dispersion of the co-amorphous system into the polymer via weak interactions as co-amorphous solid dispersions. The dissolution profile of co-amorphous NP-ST was 4.1- and 6.7-fold higher than that of crystalline NPs in pH 1.2 and 6.8 buffers, respectively. The drug concentration in the ternary amorphous system in pH 1.2 and 6.8 buffers became 1.1–1.2- and 1.4–2.7-fold higher than that seen in the co-amorphous system, respectively. **Conclusions:** Co-amorphous solid dispersion is a promising method for enhancing the solubility of brick dust molecules.

## 1. Introduction

Solubility is a vital physicochemical parameter for drug design and development. The peroral route is the most common therapeutic pathway, owing to its convenience and non-invasiveness. In fact, a drug released from a pharmaceutical dosage form via this route dissolves in the gastrointestinal medium and penetrates the intestinal membrane for absorption [1]. However, poor water solubility has become a major issue in drug discovery and development, with approximately 40% of marketed drugs and 90% of drug candidates having poor water solubility [2]. To overcome this issue, modifications of the crystal form, particle size, and formulation using various excipients have been explored.

Amorphization is a promising strategy for improving the solubility and dissolution profile of drugs as disrupting their crystal lattice can induce a higher-energy state [3]. However, this change often causes kinetic recrystallization as nucleation and crystal growth induced by molecular mobility based on a non-equilibrium state. Blending a hydrophilic polymer with an amorphous drug at the molecular level has been widely investigated as a polymeric solid dispersion formulation to stabilize the amorphous state [4]. In the dispersion of each component, the drug and polymer form intermolecular interactions such as hydrogen bonding, resulting in reduced molecular mobility. In solid dispersion formulations, hydrophilic polymers such as poly(vinylpyrrolidone-co-vinyl acetate) (VA), Soluplus^®^ (SO) and hydroxypropylmethylcellulose acetate succinate (AS) act as antiplasticizers via the formation of intermolecular interactions with amorphous drugs, resulting in the enhancement of the glass transition temperature (*Tg*) and prevention of nucleation and crystal growth. Products based on solid dispersions have been developed and released in the market using a combination of various hydrophilic polymers [5].

In recent studies, co-amorphous systems have been used as an alternative amorphous strategy and a counterpart to polymeric solid dispersions. A co-amorphous system comprises a drug molecule and a small molecule. Pharmaceutical excipients, such as amino acids, other organic acids, nicotinamide, saccharin, urea, and sugars, are major co-formers in co-amorphous systems [6]. The co-amorphous system is stabilized via miscible dispersion at the molecular level and the formation of intermolecular interactions between the drug and co-former, similar to a solid dispersion. In addition to their combination with excipients, drug–drug co-amorphous systems have emerged as a therapeutic strategy [7,8]. In a co-amorphous system without excipients, different drug molecules can be stabilized by each other via intermolecular interactions. Co-amorphous drugs have enhanced solubility and dissolution profiles with synchronized release and co-delivery to the target area [7,8,9]. However, the design of a co-amorphous system is associated with issues related to *Tg*. Most co-formers used for co-amorphous formation have a *Tg* that is less than 100 °C [10]; this temperature is lower than that of the polymeric carriers used for solid dispersion [4]. Organic acids converted to sodium salt, lactose, and taurocholic acids have been successfully applied as co-formers with a *Tg* that is greater than 100 °C [11,12,13,14].

The design of a ternary system with the addition of a hydrophilic polymer as the third component is another concern for co-amorphous systems. The addition of hydroxypropyl methylcellulose to co-amorphous carvedilol-aspartic acid/carvedilol-tryptophan were studied as ternary amorphous systems [15,16]. SO successfully formed a ternary solid dispersion with co-amorphous ezetimide–lovastatin, resulting in an improved dissolution profile and maintenance of a higher stability [17]. In other cases, by adding hydrophilic polymers, such as VA and cellulosic polymers, the compounds can become miscible with a co-amorphous system and exhibit enhanced stability and dissolution [18]. Based on these findings, ternary solid dispersions are promising systems for further improving the stability of co-amorphous systems. However, the reported cases are limited, and a brick dust molecule with a high melting point (>200 °C) and ultralow solubility (<10 μg/mL) has never been examined.

In this study, we aimed to design a ternary solid dispersion based on a co-amorphous system and assess the crystallization behavior and dissolution–permeation performance of a brick dust molecule. Antagonists of the neuropeptide Y5 receptor are known drug candidates for obesity [19]. Research into the structure–activity relationship revealed that pharmacokinetic profiles such CYP450 inhibition and oral bioavailability were improved by the modification of structure, and the inhibition of the neuropeptide Y5 receptor resulted in a reduction in body weight gain in mice via the regulation of food intake and inhibition of energy expenditure [19,20,21].

**Figure 1:**
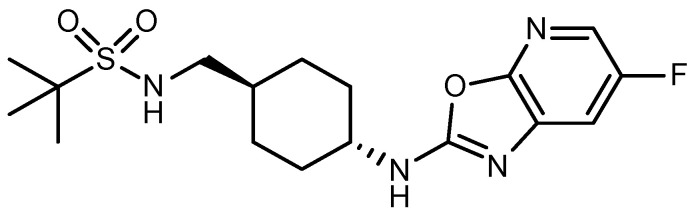
Chemical structure of antagonists of the neuropeptide Y5 receptor used in this study.

We investigated a co-amorphous formulation using a neuropeptide Y5 receptor antagonist as a brick dust drug molecule [14]. Figure 1 shows the chemical structure of the compound used in this study, and its physicochemical properties were calculated using the PhysChem Profiler Module of ACD/Percept aver. 14.3.0 (Advanced Chemistry Development, Toronto, ON, Canada). The acidic and basic pKa values were characterized for the amines next to cyclohexane and those incorporated into the sulfonamide, respectively. However, these pKa values were −0.1 (acid) and 11.7 (base), suggesting a relatively weak basic compound. The solubilities in pH 1.2 and 6.8 buffers, as well as water, were reported as 9.0, 2.4, and 2.6 μg/mL, respectively [14]. The compound was relatively soluble in the pH 1.2 buffer due to a weak basic nature, which was under 10 μg/mL. In addition, the solubilities of NPs in the pH 6.8 buffer and water were <5 μg/mL. There were two hydrogen donors and seven acceptors, suggesting the possibility of intermolecular interactions with co-formers and/or solid dispersion carriers. The ternary solid dispersion was formulated by adding a hydrophilic polymer to the co-amorphous system, and its effect on the physicochemical properties, crystallization behavior, and dissolution–permeation performance was determined.

## 2. Materials and Methods

### 2.1. Materials

*N*-(((1*r*,4*r*)-4-((6-fluorobenzo[*d*]oxazol-2-yl)amino)cyclohexyl)methyl)-2-methylpropane-2-sulfonamide, a neuropeptide Y5 receptor antagonist (NP), was synthesized by Shionogi & Co., Ltd. (Toyonaka, Osaka, Japan) [14]. Sodium taurocholate (ST) was obtained from Fujifilm Wako Pure Chemical Corporation (Chuo, Osaka, Japan). Kollidon^®^ VA64 (VA) and Soluplus^®^ (SO) were kindly provided by BASF Japan Ltd. (Chuo, Tokyo, Japan). Shin-Etsu AQOAT^®^ AS-MF (hydroxypropyl methylcellulose acetate succinate, AS) was provided by Shin-Etsu Chemical Co., Ltd. (Chiyoda, Tokyo, Japan).

A co-amorphous system comprising equal molar ratios of NPs and ST was prepared via ball milling. In a previous study, an aluminum-inserted vial was used to reduce the adhesion of the samples [14]. However, samples including polymers overflowed and were caught in the gap between the aluminum and the tube because of their low bulk density, and ball milling could not be performed. A 2 mL hard master tube (Bio Medical Science Corporation, Shinjuku, Tokyo, Japan) was used in this study; a total of 100 mg of NPs and ST was weighed in a tube containing two tungsten balls (6 mm diameter). Samples were milled using a SHAKE MASTER NEO (Bio Medical Science Corporation, Shinjuku, Tokyo, Japan) at 1500 rpm for 30 min. Ternary solid dispersions were prepared using the same method. A total of 50 mg of NPs and ST at equal molar ratios and 50 mg of each polymer were weighed in a 2 mL tube and subjected to ball milling as described above.

### 2.2. Scanning Electron Microscopy

The scanning electron microscopy (SEM) images of intact and ball-milled samples were observed using Miniscope^®^ TM3030 (Hitachi High-Technologies Corporation, Minato, Tokyo, Japan). Acceleration voltage was maintained at 15 kV during each observation period. Before sample observation, all samples were sputter-coated with gold using E-1045 sputtering equipment (Hitachi High-Technologies Corporation, Minato, Tokyo, Japan).

### 2.3. X-ray Powder Diffraction

Amorphization from the crystal form was measured using X-ray powder diffraction (XRPD) on a SmartLab (Rigaku Corporation, Akishima, Tokyo, Japan) instrument equipped with a 9 kW rotating anode with Cu Kα radiation (λ = 1.54186 Å) and a HyPix-3000 detector. The sample was placed in a hole (diameter: 3 mm; depth: 0.1 mm) on an aluminum plate and then smoothed using a spatula. The distance between the sample and detector was 331 mm, and the diffractometer was equipped with a cross-beam optical system to provide a parallel beam. By using a parallel-slit collimator with 2.5° collimation and a slit of 0.05 mm height and 0.5 mm width, the beam footprint for all measurement configurations was smaller than that of the sample. Of note, no slits were used on the receiving side. The Cu-Kα radiation point source was operated at 40 kV and 200 mA. The scan was performed from 3° to 32° (2θ) in 0.02° steps and the counting time was 40 s. β-axis rotation (20 rpm) was performed during data collection. Data were analyzed using SmartLab Studio II X64 version 4.2.111.0 (Rigaku Corporation, Akishima, Tokyo, Japan).

### 2.4. Modulated Differential Scanning Calorimetry

Modulated differential scanning calorimetry (mDSC) was performed to determine the *Tg* and crystallization point (*Tc*) using a Discovery DSC (TA Instruments Japan, Shinagawa, Tokyo, Japan). Approximately 1–5 mg of the sample was weighed in a Tzero aluminum pan and sealed with a Tzero lid. The samples were stored in a desiccator containing silica gel until measurement. Nitrogen was used as a purge gas at a flow rate of 50 mL/min. The instrument was calibrated using an indium standard. The reversing and non-reversing heat flows of the sample were obtained via heating from 0 °C to 200 °C at a heating rate of 2 °C/min. The amplitude was 0.2120 °C and the period was 40 s. The data were analyzed using Trios software version 3.3.1 (TA Instruments Japan, Shinagawa, Tokyo, Japan); the midpoint of the glass transition and the onset of the crystallization were selected as *Tg* and *Tc*, respectively.

The theoretical *Tg* of the co-amorphous system was calculated using the Gordon–Taylor (GT) Equation (1) [11].

*Tg* = (w_1_ · *Tg*_1_ + *K* · *w*_2_ · *Tg*_2_)/(*w*_1_ + *K* · *w*_2_) (1)

where *w*_1_ and *w*_2_ are the weight fractions, and *Tg*_1_ and *Tg*_2_ are the *Tg* (*K*) values of components 1 and 2, respectively. *K* was determined using Equation (2).

*K* = *Tg*_1_ · *ρ*_1_/*Tg*_2_ · *ρ*_2_ (2)

where *ρ*_1_ and *ρ*_2_ are the true densities of the components.

The modified GT Equation (3) [11] was applied to an amorphous system that comprised three components to predict the theoretical *Tg*.

*Tg* = (*w*_1_ · *Tg*_1_ + *K*_1_ · *w*_2_ · *Tg*_2_ + *K*_2_ · *w*_3_ · *Tg*_3_)/(*w*_1_ + *K*_1_ · *w*_2_ + *K*_2_ · *w*_3_) (3)

where *w*_1_, *w*_2_, and *w*_3_ are the weight fractions, and *Tg*_1_, *Tg*_2_, and *Tg*_3_ are the *Tg* (K) values of components 1, 2, and 3, respectively. *K*_1_ and *K*_2_ were determined using Equations (4) and (5).

*K*_1_ = *Tg*_1_ · *ρ*_1_/*Tg*_2_ · *ρ*_2_ (4)

*K*_2_ = *Tg*_1_ · *ρ*_1_/*Tg*_3_ · *ρ*_3_ (5)

The densities of VA (1.12 g/cm^3^) and SO (1.15 g/cm^3^) were measured using a Quantachrome Ultrapic 1200e (Anton Paar Japan K. K., Sumida, Tokyo, Japan) according to a previous method [14]. The densities of 1.35, 1.28, and 1.25 g/cm^3^ for the NPs, ST, and AS, respectively, were determined elsewhere [14,22].

### 2.5. Fourier Transform Infrared Spectroscopy

Fourier transform infrared (FT-IR) spectroscopy was performed to analyze the molecular states of the samples using a VERTEX 70 spectrometer (Bruker Optics K.K., Yokohama, Kanagawa, Japan). The samples were measured 64 times with a resolution of 4 cm^−1^ in the range of 4000–1000 cm^−1^. The peak positions were assigned using ACD/Spectrus Processor version 2019.2.2 software (Advanced Chemistry Development Inc., Toronto, ON, Canada).

### 2.6. Isothermal Crystallization Behavior

The isothermal crystallization behavior of the co-amorphous and solid dispersions was examined. The samples were stored in a desiccator with silica gel at 40 °C for 3 months. XRPD measurements were performed to confirm that the samples were in an amorphous state.

### 2.7. Dissolution Test

The dissolution profiles of the co-amorphous system and solid dispersions were obtained using a μDiss with the UV monitoring system Rainbow R6 (pION Inc., Billerica, MA, USA). Hydrochloric acid (pH 1.2) and phosphate (pH 6.8) buffers were prepared as simulated gastric and intestinal media, respectively, according to US Pharmacopoeia. Twenty milliliters of each medium was placed in each dissolution vessel and heated at 37 °C. Each sample corresponding to 2 mg of NPs was weighed in each dissolution vessel, and the dissolution test started with magnetic stirring at 300 rpm. The drug concentration was monitored using a UV probe (2 mm) inserted into the media at 295 nm (pH 1.2) and 300 nm (pH 6.8), with a baseline correction at 500 nm every 0.083 min for 0–10 min, every 0.333 min for 10–60 min, and every 1 min for 60–180 min.

### 2.8. Dynamic Laser Scattering

The number of drug-rich colloids formed in the dissolution test was measured. The samples were collected from the dissolution media using a 1 mL syringe (Terumo Corporation, Shibuya, Tokyo, Japan) and filtered using a 0.45 μm filter (GL Sciences Inc., Shinjuku, Tokyo, Japan). The filtrates were dispensed into a disposable cuvette, and dynamic light scattering (DLS) measurements were performed using a Zetasizer Nano-ZS (Malvern Panalytical Ltd., Grovewood Road, Malvern, UK). The mean diameter was calculated using photon correlation spectroscopy, and the attenuation and measurement settings were optimized automatically using the Zetasizer Software version 8.01 (Malvern Panalytical Ltd., Grovewood Road, Malvern, UK).

## 3. Results and Discussion

### 3.1. Preparation of the Amorphous Systems

Different methods have been extensively explored to prepare co-amorphous systems [6]. Solvent evaporation/spray drying from the solution state or melting–cooling processes indicate the formation of amorphous formulations. However, the NP is a brick dust molecule with ultralow solubility in aqueous media and organic solvents and a high melting point of approximately 250 °C [14]. As large volumes of organic solvent for solvent evaporation/spray drying and heating processes exceeding 250 °C are required, ball milling was employed as a mechanical process. Moreover, as ST could stabilize NPs as a co-amorphous system, as found in a previous study [14], ST was employed in this study. Therefore, ball milling was used to prepare a co-amorphous system and solid dispersions.

**Figure 2:**
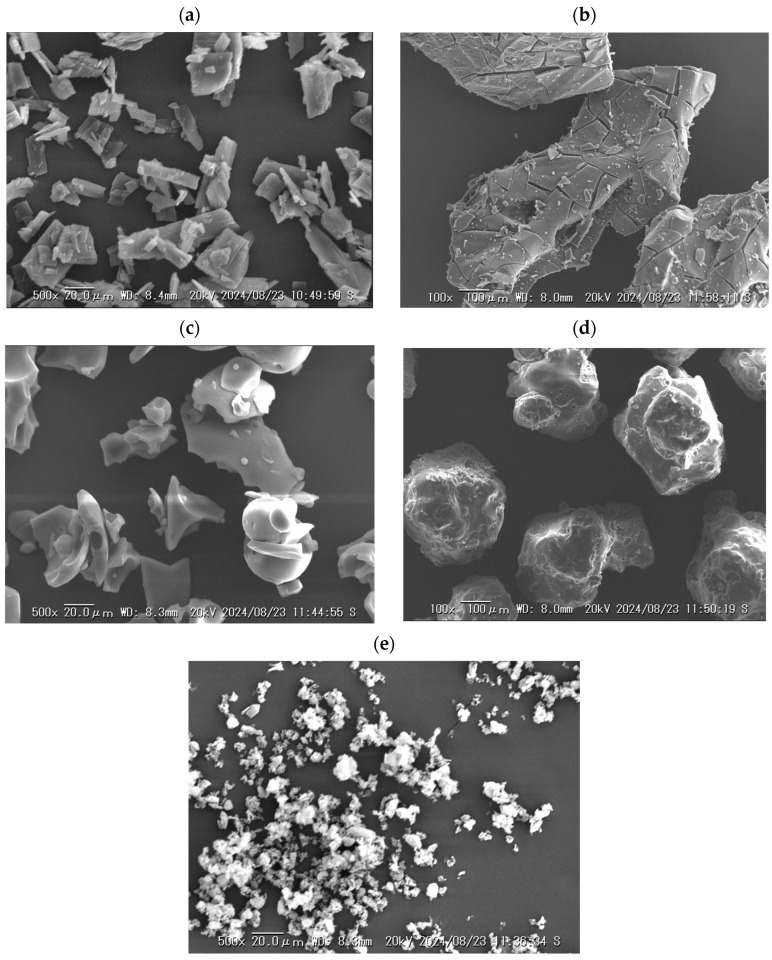
SEM images: (**a**) crystalline NPs, (**b**) ST, (**c**) VA, (**d**) SO, and (**e**) AS.

**Figure 3:**
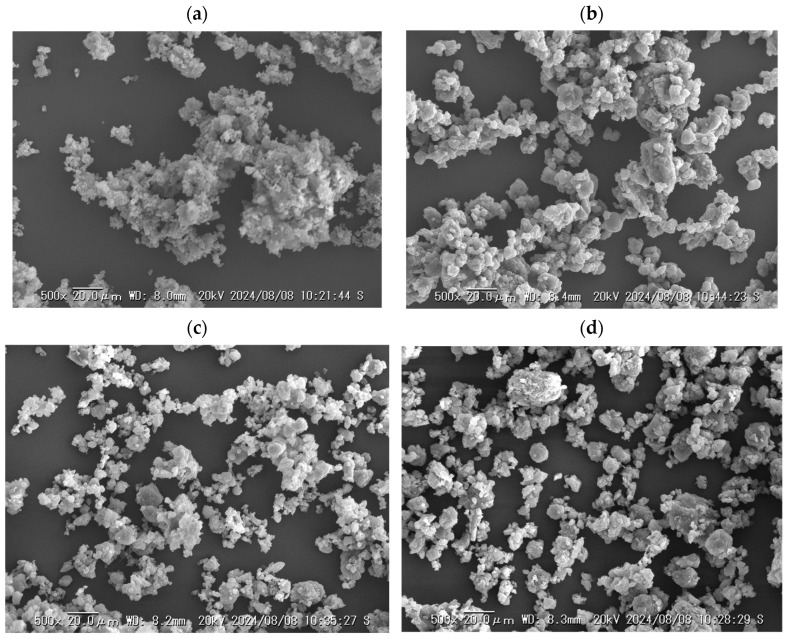
SEM images: (**a**) NP-ST, (**b**) NP-ST-VA, (**c**) NP-ST-SO, and (**d**) NP-ST-AS after ball milling.

The SEM images of NPs, ST, and the polymers are shown in Figure 2a–e. The NPs were approximately 10–50 μm in size with plate-like morphology, suggesting a crystal form. The particle sizes of ST and SO were relatively large (>100 μm), whereas those of VA and AS were approximately 20–100 μm and <20 μm, respectively. Figure 3a–d show the SEM images of the ball-milled samples. The appearance of all the samples was similar and their particle sizes were less than 20 μm, and relatively homogeneous morphologies were observed without the original particles for each component. A similar morphology and particle sizes were obtained for different drugs after ball milling with these polymers. The ball-milled tadalafil-VA/tadalafil-SO/griseofulvin-AS showed a reduction in the particle sizes (<20 μm) with homogeneous morphology, and formed a solid dispersion [23,24,25]. In the case of this study, NPs, ST and the polymers should form homogeneous blends.

**Figure 4:**
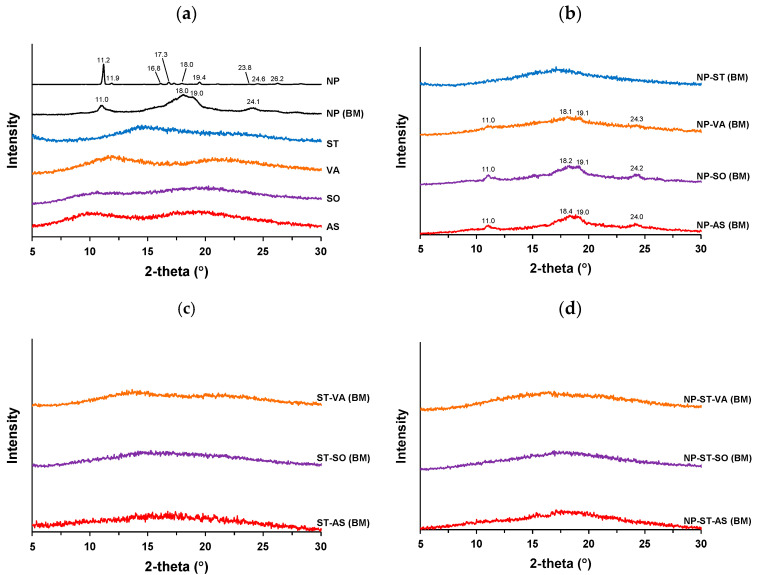
XRPD profiles: (**a**) NPs, ST, and polymers, (**b**) NP-ST and NP–polymers, (**c**) ST–polymers, and (**d**) NP-ST–polymers after ball milling (BM).

Figure 4a shows the XRPD profiles of the pure components used in this study. NPs displayed a typical X-ray diffraction pattern with peaks at 11.2°, 11.9°, 18.8°, 17.3°, 19.4°, 23.8°, 24.6, and 26.2°, aligning with the reported pattern [14]. Ball milling induced a broadening of the peaks of NP; however, the peaks did not disappear, suggesting residual crystallinity. This result agrees with that of a previous study [14]. The co-former and polymers did not have diffraction peaks, owing to their amorphous forms.

Figure 4b shows the XRPD profiles of the binary mixtures after ball milling. A halo pattern was obtained for NP-ST via ball milling, as described in a previous study [14]. ST could stabilize NPs in a co-amorphous system. However, diffraction peaks of NPs were confirmed for NP-VA, NP-SO, and NP-AS, despite the weak intensity of the peaks. This result suggests that the VA, SO, and AS polymers could not form solid dispersions, and ST had a relatively strong effect on the amorphization of NPs. The co-amorphous system and solid dispersion were comprehensively compared; however, their formabilities for the same drug were not compared [26]. In this case, ST can strongly convert crystal NPs to an amorphous form, despite the inability of representative solid dispersion carriers to perform this conversion. ST and the polymers remained amorphous after ball milling (Figure 4c). Figure 4d shows the ternary mixtures obtained after ball milling. All ternary systems (NP-ST-VA, NP-ST-SO, and NP-ST-AS) successfully formed ternary amorphous systems; the polymers did not affect the amorphization of NP-ST.

### 3.2. Thermal Analysis

**Figure 5:**
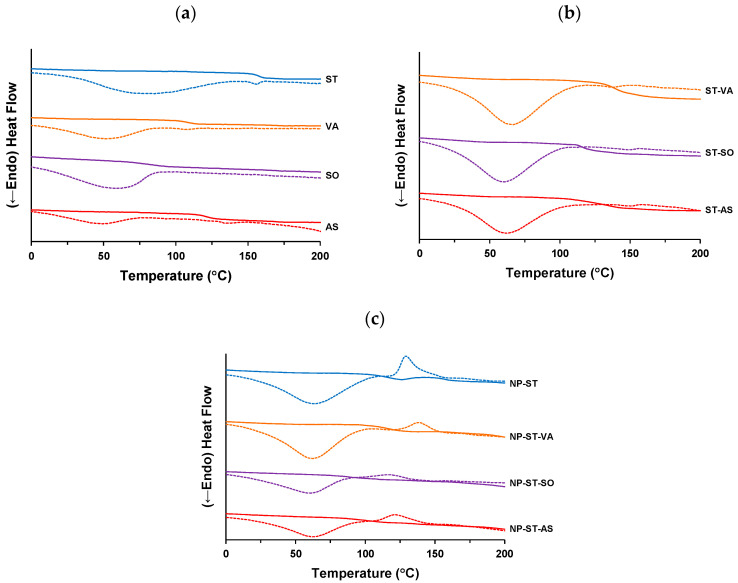
mDSC profiles: (**a**) ST and polymers, ball-milled (BM) (**b**) ST–polymer and (**c**) NP-ST/NP-ST–polymer. The solid and dashed lines represent reversing and non-reversing heat flows, respectively.

The amorphous samples prepared via ball milling were subjected to mDSC analysis. Figure 5a shows the mDSC profiles of ST and the polymers. All samples had a broadened endothermic peak in the non-reversing heat flow, suggesting the desorption of absorbed water. A typical glass transition phenomenon was observed for all excipients. The *Tg*s of ST, VA, SO, and AS were 157.5 ± 0.3 °C, 107.1 ± 1.9 °C, 80.6 ± 4.1 °C, and 121.1 ± 1.1 °C, respectively. These values are similar to the reported values for ST (155.2 °C), VA (105 °C), SO (72 °C), and AS (122 °C) [4,14].

Figure 5b shows the mDSC profiles of the ST–polymers after ball milling. Non-reversing heat flow did not cause a significant change compared with that of the components shown in Figure 5a. However, all samples had reversing heat flow profiles with single *Tg*s at 139.0 ± 0.6 °C, 114.0 ± 3.4 °C, and 135.2 ± 1.4 °C for ST-VA, ST-SO, and ST-AS, respectively. A single *Tg* is a feature of the miscible dispersion of the components in an amorphous blend [27]. If each component disperses heterogeneously, two or more *Tg* phases are induced [28]. The formation of a solid dispersion between the ST and each polymer is indicated. The comparison between the experimental and theoretical *Tg* values is focused on miscible dispersion. Table 1 presents the experimental and theoretical *Tg* values of the ball-milled samples. The theoretical *Tg* of ST-VA was 129.1 °C, with a positive deviation of approximately 10 °C from the experimental value. ST-SO and ST-AS resulted in theoretical *Tgs* of 113.3 °C and 138.3 °C, respectively, with deviations of approximately 1–3 °C from the experimental value. These differences between the experimental and theoretical *Tg*s suggest a change in the mode of intermolecular interactions, owing to the formation of a multi-amorphous system. A positive deviation of the experimental *Tg* reflects the number and/or strength of the interactions compared with that of each component. However, a negative deviation may suggest a loss in the overall interaction bonds [29]. These results suggest that VA forms a more stable solid dispersion with ST than the other components.

Figure 5c shows the mDSC profiles of the NP-ST and NP-ST–polymers after ball milling. Similar to the ST–polymers, a typical single *Tg* was observed in the reversing heat flow profiles for all samples, suggesting miscible formation of the amorphous blend. NP-ST had a *Tg* of 115.4 ± 2.4 °C, which is similar to its theoretical value (116.9 °C). The addition of an equal weight ratio of each polymer affected the reverse heat flow profile of the co-amorphous NP-ST. The *Tg* of NP-ST-VA was 114.4 ± 6.8 °C, which is similar to its theoretical value of 111.6 °C. A positive deviation was not retained for ST-VA in the ternary system of NP-ST-VA. The experimental *Tg* of NP-ST-SO was relatively low (89.5 ± 1.8 °C). SO acts as a plasticizer for NP-ST [29]. A negative deviation from the theoretical value of 96.7 °C was observed for NP-ST-SO. Furthermore, a negative deviation of approximately 19 °C was found for NP-ST-AS (experimental and theoretical *Tg*s of 100.5 ± 1.0 °C and 119.0 °C, respectively). A decrease in *Tg* is an indicator of chemical and physical instability, owing to an increase in molecular mobility [4,29]. VA can be considered a better polymer for the stability of co-amorphous NP-ST because of its highest *Tg* among the ternary amorphous systems.

The crystallization behavior appeared as exothermic peaks in the non-reversing heat flows for the co-amorphous NP-ST and ternary amorphous NP-ST–polymers (Figure 5c). The *Tc* of NP-ST was 122.0 ± 0.1 °C, with a deviation of 6.6 °C from the *Tg*. ST can stabilize NPs in a co-amorphous system; however, rapid crystallization is induced following *Tg*. NP-ST-VA had the highest *Tc* (126.7 °C among samples, with a deviation of 12.3 °C from the *Tg*). Although the *Tg* of NP-ST-SO was only 89.5 ± 1.8 °C, its *Tc* was 103.6 ± 2.4 °C, with a deviation of 14.1 °C from the *Tg*. The *Tc* of NP-ST-AS was 109.0 ± 1.2 °C, with a deviation of 8.5± 1.8 °C from the *Tg*. This value was the lowest in the ternary amorphous system, suggesting that the stabilizing effect of AS on co-amorphous NP-ST was relatively lower than that of VA and SO.

### 3.3. Spectroscopic Analysis

**Figure 6:**
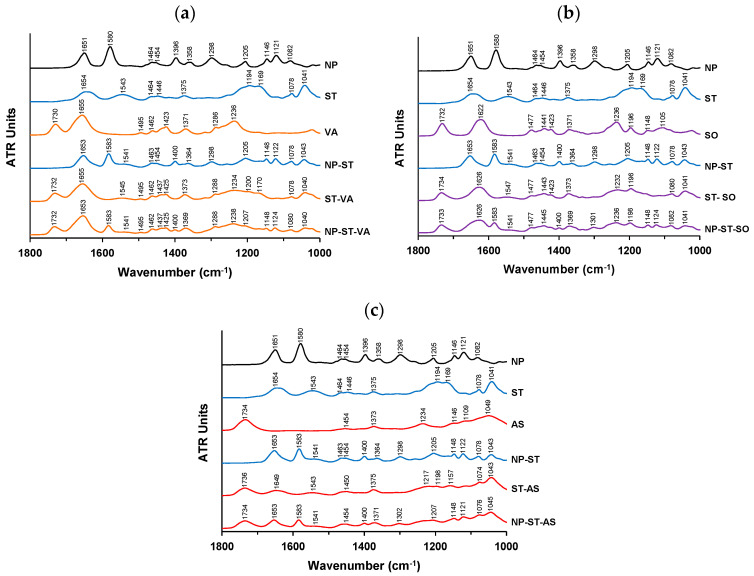
Infrared spectra of amorphous NPs, ST, polymers, co-amorphous NP-ST, and ternary amorphous blends of NP-ST–polymers, including (**a**) VA, (**b**) SO, and (**c**) AS. Amorphous NP data obtained in a previous study [14] were used.

To determine the intermolecular interactions in co-amorphous NP-ST and ternary amorphous systems, the NP data obtained in a previous study [14] were used. Figure 6a shows the FTIR spectra of amorphous NPs, ST, and VA, co-amorphous NP-ST, solid dispersion ST-VA, and ternary amorphous NP-ST-VA. The NP and ST peaks were assigned according to a previous study [14]. The peaks of the NPs at 1651 cm^−1^ and 1580 cm^−1^ represent a C=N stretching of oxazolopyridine and N-H_2_ in the plane band, respectively. CH_3_ deformations of the tertiary butyl group, the S=O stretching of sulfonamide, the C-O-C stretching of oxazolopyridine, and C-F stretching were identified at 1396 cm^−1^, 1358 cm^−1^, 1298 cm^−1^, and 1205 cm^−1^, respectively. C-H bending of oxazolopyridine is indicated by peaks at 1146 cm^−1^ and 1121 cm^−1^. ST exhibited relatively broad peaks in its spectrum. In the ST spectrum, the C=O stretching of amide I at 1654 cm^−1^ and the S=O stretching of sulfonate at 1194/1169 cm^−1^ were characterized. The spectrum of VA was compared to that obtained previously [30]; the typical C=O stretching was indicated by peaks at 1730 cm^−1^ and 1655 cm^−1^.

Most of the peaks in the NP and ST samples were observed at similar positions in the spectrum of the co-amorphous NP-ST. However, the peak at 1358 cm^−1^ assigned to the S=O stretching of sulfonamide in the NPs had a 6 cm^−1^ shift to 1364 cm^−1^. In addition, the peak at 1194/1169 cm^−1^ for the S=O stretching of sulfonate derived from ST almost disappeared. These results suggest the formation of intermolecular interactions. In the spectrum of ST-VA, the peak at 1194 cm^−1^ for the S=O stretching of sulfonate shifted to 1200 cm^−1^, suggesting an interaction with VA, despite no changes in the peaks of VA. Similar changes were observed in the NP-ST-VA spectrum with the addition of VA to co-amorphous NP-ST. The peak at 1364 cm^−1^ for the S=O stretching of sulfonamide in the NPs merged with the peak at 1371 cm^−1^ in VA and appeared at 1369 cm^−1^, suggesting a retention of the intermolecular interaction of NP-ST in the ternary system. The peak at 1194/1169 cm^−1^ for the S=O stretching of sulfonate in ST almost disappeared. This result implies that the intermolecular interaction between the NP and ST in the co-amorphous system was maintained in the ternary system with VA, and no strong interaction was formed between the NP and VA/ST and VA.

Figure 6b and c show the FT-IR spectra of each component and the amorphous systems, including SO and AS, respectively. The peaks of ST, SO, and AS appeared at similar positions in the solid dispersions of ST-SO and ST-AS, respectively, except for the S=O stretching of the sulfonate of ST at 1194/1169 cm^−1^. The peak at 1169 cm^−1^ almost disappeared following the addition of SO, and shifted to 1157 cm^−1^ in the spectrum of ST-AS. The sulfonate of ST interacted with the polymers. The peak at 1358/1364 cm^−1^ for the S=O stretching of sulfonamide in the spectra of amorphous ST/co-amorphous NP-ST further shifted to 1369 cm^−1^ and 1371 cm^−1^ in the ternary systems of NP-ST-SO and NP-ST-AS, respectively. The intermolecular interactions between the NP and ST were maintained in dispersions with SO or AS.

The intermolecular interactions of NP-ST as a co-amorphous system were maintained in all ternary amorphous systems. The deviation of the experimental from the theoretical *Tg*s (Table 1) indicates that the S=O of the sulfonamide of the NP leads to the formation of intermolecular interactions with polymers according to the strength of AS > SO > VA. A case of no intermolecular interactions in the FT-IR spectra for a multi-amorphous system has been reported. Simvastatin formed a co-amorphous system with glibenclamide via ball milling. No intermolecular interaction was detected in the FT-IR spectra of the co-amorphous system; however, an improved stability and dissolution rate were achieved, owing to the miscible dispersion of components. Moreover, the deviation of the experimental *Tg* from the theoretical *Tg* implied a weak interaction [31]. Another study revealed that the addition of a cellulosic polymer or VA to co-amorphous carvedilol–tryptophan disrupted the intermolecular interactions between the drug and co-former, and confirmed the formation of alternative interactions between the drug and polymer [32]. This finding suggests a miscible dispersion of co-amorphous NP-ST with maintenance of its intermolecular interactions in dispersion with each polymer as a co-amorphous solid dispersion.

### 3.4. Isothermal Crystallization Behavior

**Figure 7:**
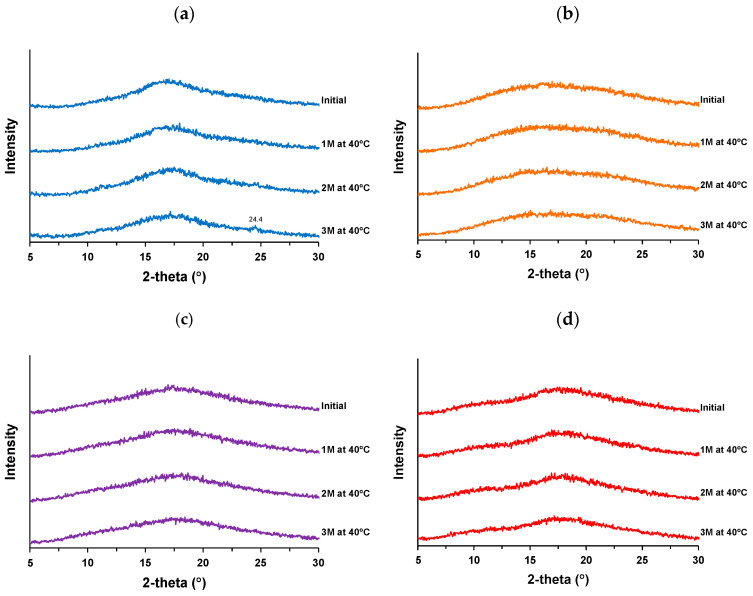
XRPD profiles of (**a**) co-amorphous NP-ST and co-amorphous solid dispersions (**b**) NP-ST-VA, (**c**) NP-ST-SO, and (**d**) NP-ST-AS before and after the stability test at 40 °C with silica gel for 3 months.

The isothermal crystallization behavior at 40 °C was examined for the co-amorphous NP-ST and the ternary systems of NP-ST–polymers. Figure 7 a, b, c, and d show the XRPD profiles of NP-ST, NP-ST-VA, NP-ST-SO, and NP-ST-AS before and after storage at 40 °C for 3 months, respectively. No crystalline peaks were observed in any of the samples at 1 and 2 months; however, the co-amorphous NP-ST had a small diffraction peak at 24.4° after 3 months (Figure 7a). This peak was observed in the profile of the NP after ball milling (Figure 4a), suggesting the crystallization of the NP during storage. All ternary amorphous systems maintained their amorphous forms and prevented the crystallization of NPs.

The formation of intermolecular interactions with polymeric carriers and the reduction in molecular mobility by an increase in *Tg* are well known as the major mechanisms for stabilizing solid dispersions [33]. In this study, the *Tg* of NP-ST was comparable to that of NP-ST-VA and higher than that of NP-ST-SO and NP-ST-AS, despite a higher deviation between *Tg* and *Tc* for the ternary systems (Table 1). The possibility of weak intermolecular interactions between the polymer and the NP is discussed in Section 3.3, Spectroscopic Analysis, and implies its ability to stabilize co-amorphous solid dispersions.

### 3.5. Dissolution Test

**Figure 8:**
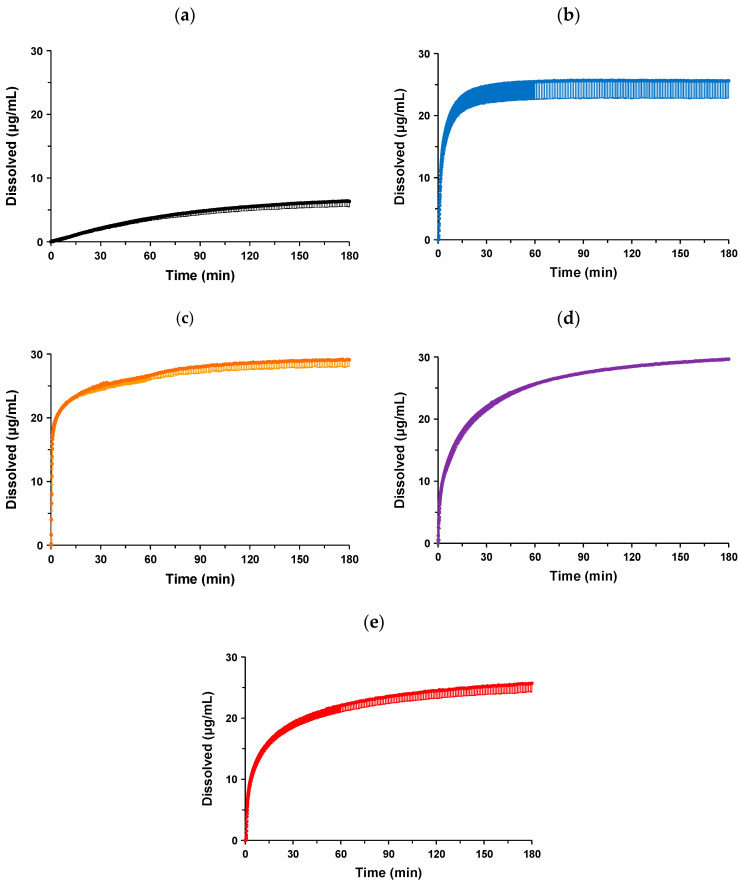
Dissolution profiles of (**a**) crystalline NPs, (**b**) co-amorphous NP-ST, and co-amorphous solid dispersions (**c**) NP-ST-VA, (**d**) NP-ST-SO, and (**e**) NP-ST-AS in hydrochloric buffer (pH 1.2). The error bars represent the standard deviation of n = 3.

**Figure 9:**
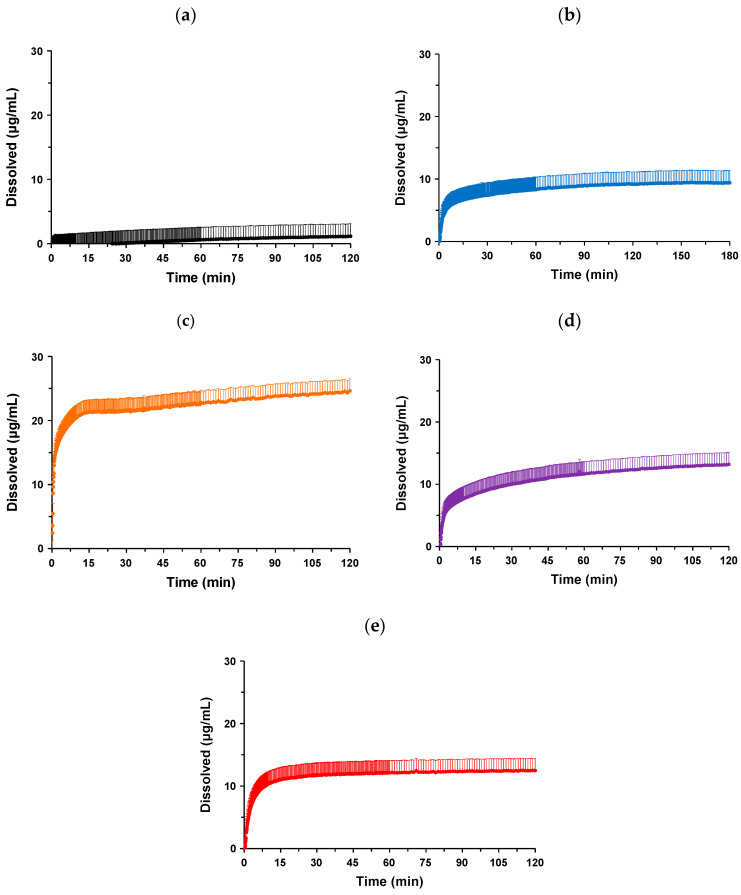
Dissolution profiles of (**a**) crystalline NPs, (**b**) co-amorphous NP-ST, and co-amorphous solid dispersions (**c**) NP-ST-VA, (**d**) NP-ST-SO, and (**e**) NP-ST-AS in phosphate buffer (pH 6.8). The error bars represent the standard deviation of n = 3.

**Figure A1:**
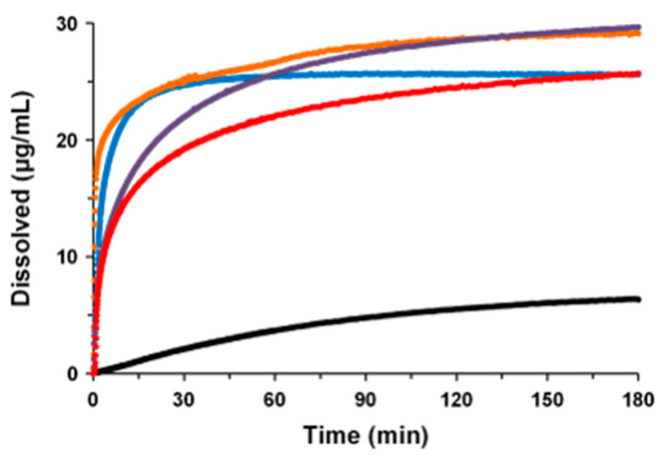
Dissolution profiles in hydrochloric buffer (pH 1.2) shown in Figure 8a–e without the error bars (black, crystalline NP; blue, co-amorphous NP-ST; co-amorphous solid dispersions: orange, NP-ST-VA; purple, NP-ST-SO; red, NP-ST-AS).

**Figure A2:**
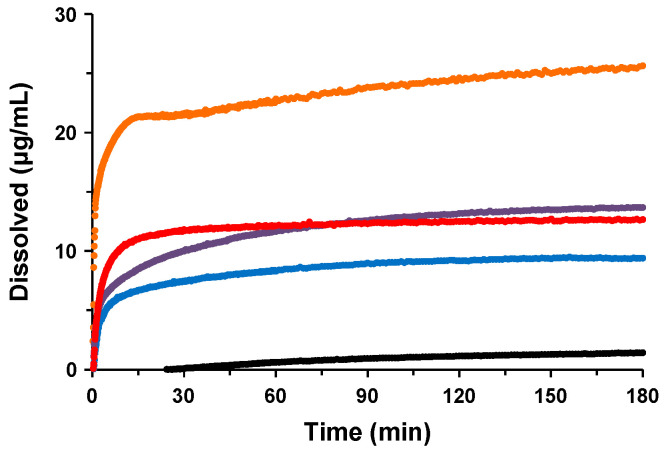
Dissolution profiles in phosphate buffer (pH 6.8) shown in Figure 9a–e without the error bars (black, crystalline NP; blue, co-amorphous NP-ST; co-amorphous solid dispersions: orange, NP-ST-VA; purple, NP-ST-SO; red, NP-ST-AS).

The dissolution profiles of the co-amorphous system and co-amorphous solid dispersions in hydrochloric (pH 1.2) and phosphate (pH 6.8) buffers are compared in Figure 8a–e and Figure 9a–e, respectively. The summarized data without error bars are shown in Figure A1 and Figure A2. In the hydrochloric buffer (pH 1.2), crystalline NPs showed a slow dissolution and reached only approximately 6.3 ± 0.9 μg/mL at 180 min, which was similar to the solubility data [14]. A rapid dissolution of NP-ST was observed immediately after the initiation of the experiment, with a concentration of approximately 24.7 ± 2.7 μg/mL achieved at 30 min, followed by a gradual increase to approximately 25.6 ± 2.8 μg/mL at 180 min. ST induced a 4.1-fold increase in the dissolution profile of the NP in the hydrochloric buffer (pH 1.2) through the formation of a co-amorphous system.

The addition of hydrophilic polymers affected the dissolution profile in the hydrochloric buffer (pH 1.2). In the case of NP-ST-VA, both the most rapid drug release and improvement in the dissolution corresponded to 29.1 ± 1.0 μg/mL at 180 min. Although the initial drug release of the ternary NP-ST-SO system was delayed compared to that of the co-amorphous system, 29.7 ± 0.2 μg/mL at 180 min was superior to that of NP-ST. SO is a hydrophilic solid dispersion carrier; however, its chemical structure comprises vinyl acetate and vinylcaprolactam moieties as hydrophobic parts, in addition to a hydrophilic backbone [34]. The relatively low wettability of SO, which delays the release of NPs, has been characterized in solid dispersions with griseofulvin [35]. NP-ST-AS showed the slowest drug release among all amorphous formulations. It is well known that AS is an enteric polymer caused by the incorporated substitution of hydroxypropoxy, methoxy, acetyl, and succinoyl groups with a cellulosic backbone [36]. The insolubility of AS in an acidic buffer should induce a delay in drug release and not improve the maximum concentration of the co-amorphous system.

The dissolution profiles of the NP and formulations in the phosphate buffer (pH 6.8) showed different tendencies. Crystalline NPs were not released until approximately 24 min, followed by a slow dissolution, and reached only 1.4 ± 2.0 μg/mL at 180 min. As discussed in Section 1, Introduction, the weak basic nature of NPs should cause a drastic reduction in drug release and maximum concentration in the phosphate buffer (pH 6.8) compared to those in the hydrochloric buffer (pH 1.2). The formation of a co-amorphous system with ST resulted in a remarkable improvement in the dissolution profile. Rapid drug release was achieved, reaching 6.1 ± 2.1 μg/mL in 9 min. The concentration at 180 min was 9.4 ± 2.8 μg/mL. The improvement effect of the co-amorphization with ST on the dissolution profile of the NP was particularly characterized in the phosphate buffer (pH 6.8). The dissolution of the crystalline NP was less than 5 μg/mL at 180 min, and co-amorphization with ST induced an approximately 6.7-fold improvement in the concentration. The nucleation and precipitation of amorphous drugs during dissolution tests are well known. The higher-energy state of the amorphous form induces the quick release of the drug to a supersaturated concentration and rapid precipitation corresponding to the spring pattern. Hydrophilic polymers and co-formers can delay the precipitation of supersaturated drugs in solid dispersions and co-amorphous systems, respectively [4,6]. ST acted as a stabilizer for supersaturated NPs in both simulated gastric and intestinal media.

The initial drug release from NP-ST-SO was found to be comparable to that of NP-ST; however, the drug concentration increased over time and was superior to that of NP-ST at approximately 5 min. The final NP-ST-SO concentration after 180 min was 13.7 ± 1.2 μg/mL. Both VA and AS are representative hydrophilic solid dispersion carriers that cause a more rapid dissolution of the NP compared with NP-ST-SO. The drug concentration of NP-ST-AS almost plateaued (11.8 ± 1.3 μg/mL) at 30 min, and the concentration at 180 min was 12.7 ± 1.1 μg/mL. On the other hand, the concentration of NP-ST-VA increased after 30 min and reached 25.6 ± 1.7 μg/mL at 180 min, which was the highest among the co-amorphous and ternary systems. AS is an enteric polymer in which solid dispersion sometimes causes the slow dissolution of drugs in neutral media [36]. The formulation of carbamazepine solid dispersion using VA or AS improved the initial drug release and final dissolution when compared to those of the crystalline drug, and the performance of a formulation combined with VA was superior to that using AS [37]. The most rapid release of the NP might have caused the highest dissolution of NP-ST-VA based on the higher wettability of VA.

Liquid–liquid phase separation is a concern in the mechanism of nucleation and crystallization of supersaturated drugs. Amorphous drugs dissolve in aqueous media and form a supersaturated state, resulting in a drug-rich phase. The particle size of the drug-rich colloids increases depending on the drug concentration and these colloids become unstable [38,39,40,41,42]. The distribution of a solid dispersion carrier, such as AS, into the colloids reduced the droplet size, prevented nucleation–crystallization, and improved membrane permeability and oral absorption [39,40,41,42]. For the co-amorphous solid dispersion used in this study, the blended polymers can be implied to be distributed into colloids of NPs. Dynamic laser scattering analysis was performed to determine the droplet size of the NPs in the samples after the dissolution test. However, the zeta average (nm) could not be correctly obtained, owing to the low intensity and high polydispersity index. The ultralow solubility of NPs may have also hindered our efforts for the zeta average. The higher crystallization potency of NPs may cause the rapid growth of drug-rich colloids over the nanometer range and precipitation [41,42,43]. In addition, the ternary systems contained both ST and polymers, resulting in a complex-solution phase. In reported cases, liquid–liquid phase separation from a supersaturated state was investigated using a binary system containing a drug and a hydrophilic polymer [39,40,41]. In this study, both ST and polymers may have affected the drug-rich colloids, resulting in higher polydispersity with a heterogeneous dispersion of colloids.

By adding a drug solution prepared using an organic solvent to an aqueous medium, different concentrations of a supersaturated solution can be used to examine drug-rich colloids [41,42]. To obtain a relatively higher concentration of NPs, the supersaturated state was prepared at 500 μg/mL via a 10-fold dilution of a DMSO solution of NPs at 5 mg/mL in buffer (pH 6.8) at 37 °C with or without an ST or ST–polymer with the same composition of co-amorphous system/ternary amorphous systems. However, all diluted solutions precipitated within minutes and could not be subjected to DLS measurements, despite the presence of the ST–polymer, owing to the rapid crystallization potency of NPs. An alternative method to reveal the mechanism of liquid–liquid phase separation of a brick dust molecule combined with a co-former and polymeric third components in a supersaturated state is required in future studies.

## 4. Conclusions

This study aimed to determine the effect of adding a polymer to a co-amorphous system containing NPs as a brick dust drug with ultralow solubility (<5 μg/mL). Ball milled NPs and ST converted this drug into a co-amorphous system, but not a binary solid dispersion with VA/SO/AS. The addition of each polymer to the co-amorphous NP-ST induced the formation of a co-amorphous solid dispersion following ball milling. Single *Tg*s appeared for all amorphous systems, and a small deviation from the theoretical *Tg*s was observed, except for NP-ST-AS. FT-IR spectra revealed the intermolecular interactions of NP-ST in the co-amorphous system. These interactions were characterized in ternary amorphous systems with polymers, suggesting a miscible dispersion of the co-amorphous system into the polymers via weak interactions. Blended polymers stabilized co-amorphous NP-ST at 40 °C despite crystallization without polymers. Co-amorphous NP-ST showed a 4.1- and 6.7-fold enhancement of the dissolution profile in pH 1.2 and 6.8 media; however, VA, SO, and AS further enhanced by 1.1–1.2- and 1.4–2.7-fold the final dissolution, respectively. The improvement in the dissolution profile by the formation of a co-amorphous or co-amorphous solid dispersion was better characterized in the pH 6.8 buffer due to the weakly basic nature of the NP. Notably, the greatest improvement in the dissolution profile was observed with VA. Altogether, the formulation of a co-amorphous solid dispersion without disrupting the intermolecular interactions between the drug and co-former is a reasonable approach to improve both the stability and dissolution of brick dust molecules. Further investigations will be conducted to reveal the detailed mechanism of the polymer’s effect on the dissolution performance of co-amorphous materials.

## Figures and Tables

**Table 1:** Experimental and theoretical *Tg*s of the ball-milled samples; the theoretical values were calculated using the Gordon–Taylor equation. The error bars represent the standard deviation of n = 3.

	Experimental *Tg*	Theoretical *Tg*
ST-VA	139.0 ± 0.6 °C	129.1 °C
ST-SO	114.0 ± 3.4 °C	113.3 °C
ST-AS	135.2 ± 1.4 °C	138.3 °C
NP-ST	115.4 ± 6.8 °C	116.9 °C
NP-ST-VA	114.4 ± 6.8 °C	111.6 °C
NP-ST-SO	89.5 ± 1.8 °C	96.7 °C
NP-ST-AS	100.5 ± 1.9 °C	119.0 °C

## Data Availability

The raw data presented in this article are available upon request.

## References

[1] Kumari L., Choudhari Y., Patel P., Gupta G.D., Singh D., Rosenholm J.M., Bansal K.K., Kurmi B.D. (2023). Advancement in solubilization approaches: A step towards bioavailability enhancement of poorly soluble drugs. Life.

[2] Liu X., Zhao L., Wu B., Chen F. (2023). Improving solubility of poorly water-soluble drugs by protein-based strategy: A review. Int. J. Pharm..

[3] Shi Q., Li F., Yeh S., Wang Y., Xin J. (2020). Physical stability of amorphous pharmaceutical solids: Nucleation, crystal growth, phase separation and effects of the polymers. Int. J. Pharm..

[4] Zhang J., Guo M., Luo M., Cai T. (2023). Advances in the development of amorphous solid dispersions: The role of polymeric carriers. Asian. J. Pharm. Sci..

[5] Moseson D.E., Tran T.B., Karunakaran B., Ambardekar R., Hiew T.N. (2024). Trends in amorphous solid dispersion drug products approved by the U.S. Food and Drug Administration between 2012 and 2023. Int. J. Pharm. X.

[6] Liu J., Grohganz H., Löbmann K., Rades T., Hempel N.J. (2021). Co-amorphous drug formulations in numbers: Recent advances in co-amorphous drug formulations with focus on co-formability, molar ratio, preparation methods, physical stability, in vitro and in vivo performance, and new formulation strategies. Pharmaceutics.

[7] Shelke R., Velagacherla V., Nayak U.Y. (2024). Recent advances in dual-drug co-amorphous systems. Drug Discov. Today.

[8] Wang H., Zhao P., Ma R., Jia J., Fu Q. (2024). Drug-drug co-amorphous systems: An emerging formulation strategy for poorly water-soluble drugs. Drug Discov. Today.

[9] Ueda H., Hirakawa Y., Miyano T., Nakayama Y., Hatanaka Y., Uchiyama H., Tozuka Y., Kadota K. (2023). Improvement in inhalation properties of theophylline and levofloxacin by co-amorphization and enhancement in its stability by addition of amino acid as a third component. Mol. Pharm..

[10] Yarlagadda D.L., Sai Krishna Anand V., Nair A.R., Navya Sree K.S., Dengale S.J., Bhat K. (2021). Considerations for the selection of co-formers in the preparation of co-amorphous formulations. Int. J. Pharm..

[11] Ueda H., Wu W., Löbmann K., Grohganz H., Müllertz A., Rades T. (2018). Application of a salt coformer in a co-amorphous drug system dramatically enhances the glass transition temperature: A case study of the ternary system carbamazepine, citric acid, and l-arginine. Mol. Pharm..

[12] Ueda H., Bøtker J.P., Edinger M., Löbmann K., Grohganz H., Müllertz A., Rades T., Østergaard J. (2020). Formulation of co-amorphous systems from naproxen and naproxen sodium and in situ monitoring of physicochemical state changes during dissolution testing by Raman spectroscopy. Int. J. Pharm..

[13] Ueda H., Hirakawa Y., Miyano T., Imono M., Tse J.Y., Uchiyama M., Tozuka Y., Kadota K. (2022). Design of a stable coamorphous system using lactose as an antiplasticizing agent for diphenhydramine hydrochloride with a low glass transition temperature. Mol. Pharm..

[14] Aikawa S., Tanaka H., Ueda H., Maruyama M., Higaki K. (2022). Formation of a stable co-amorphous system for a brick dust molecule by utilizing sodium taurocholate with high glass transition temperature. Pharmaceutics.

[15] Liu J., Grohganz H., Rades T. (2020). Influence of polymer addition on the amorphization, dissolution and physical stability of co-amorphous systems. Int. J. Pharm..

[16] Wang Y., Rades T., Grohganz H. (2023). Considerations on the Kinetic Processes in the Preparation of Ternary Co-Amorphous Systems by Milling. Pharmaceutics..

[17] Riekes M.K., Engelen A., Appeltans B., Rombaut P., Stulzer H.K., Van den Mooter G. (2016). New perspectives for fixed dose combinations of poorly water-soluble compounds: A case study with ezetimibe and lovastatin. Pharm. Res..

[18] Saberi A., Kouhjani M., Yari D., Jahani A., Asare-Addo K., Kamali H., Kokhodchi A. (2023). Development, recent advances, and updates in binary, ternary co-amorphous systems, and ternary solid dispersions. J. Drug Deliv. Sci. Technol..

[19] Fukasaka Y., Nambu H., Tanioka H., Obata A., Tonomura M., Okuno T., Yukioka H. (2018). An insurmountable NPY Y5 receptor antagonist exhibits superior anti-obesity effects in high-fat diet-induced obese mice. Neuropeptides.

[20] Tamura Y., Omori N., Kouyama N., Nishiura Y., Hayashi K., Watanabe K., Tanaka Y., Chiba T., Yukioka H., Sato H. (2012). Design, synthesis and identification of novel benzimidazole derivatives as highly potent NPY Y5 receptor antagonists with attractive in vitro ADME profiles. Bioorg. Med. Chem. Lett..

[21] Tamura Y., Hayashi K., Omori N., Nishiura Y., Watanabe K., Tanaka N., Fujioka M., Kouyama N., Yukimasa A., Tanaka Y. (2013). Identification of a novel benzimidazole derivative as a highly potent NPY Y5 receptor antagonist with an anti-obesity profile. Bioorg. Med. Chem. Lett..

[22] Ueda H., Hirakawa Y., Tanaka H., Miyano T., Sugita K. (2021). Applicability of an experimental grade of hydroxypropyl methylcellulose acetate succinate as a carrier for formation of solid dispersion with indomethacin. Pharmaceutics.

[23] Wlodarski K., Tajber L., Sawicki W. (2016). Physicochemical properties of direct compression tablets with spray dried and ball milled solid dispersions of tadalafil in PVP-VA. Eur. J. Pharm. Biopharm..

[24] Krupa A., Descamps M., Willart J.F., Strach B., Wyska E., Jachowicz R., Danède F. (2016). High-Energy Ball Milling as Green Process to Vitrify Tadalafil and Improve Bioavailability. Mol. Pharm..

[25] Al-Obaidi H., Lawrence M.J., Shah S., Moghul H., Al-Saden N., Bari F. (2013). Effect of drug-polymer interactions on the aqueous solubility of milled solid dispersions. Int. J. Pharm..

[26] Vullendula S.K.A., Nair A.R., Yarlagadda D.L., Navya Sree K.S., Bhat K., Dengale S.J. (2022). Polymeric solid dispersion Vs co-amorphous technology: A critical comparison. J. Drug Deliv. Sci. Technol..

[27] Chieng N., Teo X., Cheah M.H., Choo M.L., Chung J., Hew T.K., Keng P.S. (2019). Molecular dynamics and physical stability of pharmaceutical co-amorphous systems: Correlation between structural relaxation times Measured by Kohlrausch-Williams-Watts with the width of the glass transition temperature (ΔTg) and the onset of crystallization. J. Pharm. Sci..

[28] Mizoguchi R., Waraya H., Hirakura Y. (2019). Application of co-amorphous technology for improving the physicochemical properties of amorphous formulations. Mol. Pharm..

[29] Karagianni A., Kachrimanis K., Nikolakakis I. (2018). Co-amorphous solid dispersions for solubility and absorption improvement of drugs: Composition, preparation, characterization and formulations for oral delivery. Pharmaceutics.

[30] Liu C., Chen Z., Chen Y., Lu J., Li Y., Wang S., Wu G., Qian F. (2016). Improving oral bioavailability of sorafenib by optimizing the “spring” and “parachute” based on molecular interaction mechanisms. Mol. Pharm..

[31] Löbmann K., Strachan C., Grohganz H., Rades T., Korhonen O., Laitinen R. (2012). Co-amorphous simvastatin and glipizide combinations show improved physical stability without evidence of intermolecular interactions. Eur. J. Pharm. Biopharm..

[32] Wang Y., Grohganz H., Rades T. (2022). Effects of polymer addition on the non-strongly interacting binary co-amorphous system carvedilol-tryptophan. Int. J. Pharm..

[33] Al-Japairai K., Hamed Almurisi S., Mahmood S., Madheswaran T., Chatterjee B., Sri P., Mazlan N.A.B.A., Hagbani T.I., Alheibshy F. (2023). Strategies to improve the stability of amorphous solid dispersions in view of the hot melt extrusion (HME) method. Int. J. Pharm..

[34] Attia M.S., Elshahat A., Hamdy A., Fathi A.M., Emad-Eldin M., Ghazy F.E.S., Chopra H., Ibrahim T.M. (2023). Soluplus^®^ as a solubilizing excipient for poorly water-soluble drugs: Recent advances in formulation strategies and pharmaceutical product features. J. Drug Deliv. Sci. Technol..

[35] Rahman M., Ahmad S., Tarabokija J., Parker N., Bilgili E. (2020). Spray-dried amorphous solid dispersions of griseofulvin in HPC/Soluplus/SDS: Elucidating the multifaceted impact of SDS as a minor component. Pharmaceutics.

[36] Butreddy A. (2022). Hydroxypropyl methylcellulose acetate succinate as an exceptional polymer for amorphous solid dispersion formulations: A review from bench to clinic. Eur. J. Pharm. Biopharm..

[37] Yu D., Li J., Wang H., Pan H., Li T., Bu T., Zhou W., Zhang X. (2022). Role of polymers in the physical and chemical stability of amorphous solid dispersion: A case study of carbamazepine. Eur. J. Pharm. Sci..

[38] Qian K., Stella L., Jones D.S., Andrews G.P., Du H., Tian Y. (2021). Drug-rich phases induced by amorphous solid dispersion: Arbitrary or intentional goal in oral drug delivery?. Pharmaceutics.

[39] Nunes P.D., Pinto J.F., Henriques J., Paiva A.M. (2022). Insights into the release mechanisms of ITZ:HPMCAS amorphous solid dispersions: The role of drug-rich colloids. Mol. Pharm..

[40] Stewart A.M., Grass M.E., Brodeur T.J., Goodwin A.K., Morgen M.M., Friesen D.T., Vodak D.T. (2017). Impact of drug-rich colloids of itraconazole and HPMCAS on membrane flux in vitro and oral bioavailability in rats. Mol. Pharm..

[41] Ueda K., Takemoto S., Higashi K., Moribe K. (2024). Impact of colloidal drug-rich droplet size and amorphous solubility on drug membrane permeability: A comprehensive analysis. J. Pharm. Sci..

[42] Ueda K., Higashi K., Moribe K. (2019). Mechanistic elucidation of formation of drug-rich amorphous nanodroplets by dissolution of the solid dispersion formulation. Int. J. Pharm..

[43] Ueda K., Taylor L.S. (2020). Polymer type impacts amorphous solubility and drug-rich phase colloidal stability: A mechanistic study using nuclear magnetic resonance spectroscopy. Mol. Pharm..

